# Comparison of surgical outcomes among open, laparoscopic, and robotic pancreatoduodenectomy: a single-center retrospective study

**DOI:** 10.1186/s12893-022-01797-4

**Published:** 2022-09-22

**Authors:** Wei Guo, Xiaofei Ye, Jiangfa Li, Shiliu Lu, Ming Wang, Zefeng Wang, Jianni Yao, Shuiping Yu, Guandou Yuan, Songqing He

**Affiliations:** 1 Division of Hepatobiliary Surgery, The First Affiliated Hospital of Guangxi Medical University, Nanning, 530021 Guangxi China; 2 Key Laboratory of Early Prevention and Treatment for Regional High Frequency Tumor (Guangxi Medical University), Ministry of Education, Nanning, 530021 Guangxi China; 3 Guangxi Key Laboratory of Immunology and Metabolism for Liver Diseases, Nanning, 530021 Guangxi China

**Keywords:** Robotic surgery, Minimal invasive surgery, Pancreaticoduodenectomy, Complications

## Abstract

**Background:**

There is no general consensus on the feasibility and safety of robotic pancreatoduodenectomy (RPD) and whether it increases surgical risks. The purpose of this study was to assess the safety, feasibility, and rationality of RPD by comparing perioperative data among open pancreatoduodenectomy (OPD), laparoscopic pancreatoduodenectomy (LPD), and RPD performed in our center in recent years.

**Methods:**

Clinical data of patients had undergone RPD (n = 32), LPD (n = 21), and OPD (n = 86) in The First Affiliated Hospital of Guangxi Medical University between January 2016 and June 2020 were retrospectively collected and analyzed.

**Results:**

RPD required more time for operation (537.2 min vs. 441.5 min, p < 0.001) than OPD did, but less time to remove abdominal drainage tube (12.5 d vs. 17.3 d, p = 0.001). The differences between the RPD group and LPD group were interesting, as the two groups had similar operation time (537.2 min vs. 592.9 min, p = 1.000) and blood loss (482.8 ml vs. 559.5 ml, p > 0.05), but the RPD group had a higher activity of daily living score on postoperative day 3 (35.8 vs. 25.7, p = 0.0017) and a lower rate of conversion to OPD (6.5% vs. 38.1%, p = 0.011). Regarding complications, such as the postoperative pancreatic fistula, abdominal hemorrhage, intra-abdominal infection, bile leakage, reoperation, and perioperative mortality, there were no significant differences among the three groups.

**Conclusions:**

Not only is RPD feasible and reliable, it also offers significant advantages in that it improves postoperative recovery of skills needed for everyday life, has a low conversion rate to open surgery, and does not increase surgical risks.

## Introduction

Pancreatoduodenectomy (PD) was first officially reported by Whipple in 1935 [1]. Since then, PD has been progressively acknowledged as a standard procedure for removing benign and malignant tumors from the head of pancreas and the periampullary region. The convoluted procedure involves broad resection and reconstruction of the digestive tract; therefore, it has invariably been identified as one of the most complicated and risky operations.

Laparoscopic pancreatoduodenectomy (LPD)has been developed for approximately 27 years since this technology was reported by Gagner and Pomp for the first time in 1994 [2]. It is still controversial in the academic community because of its initially unacceptable feasibility and security. In recent years, LPD has been shown to provide obvious advantages such as less trauma, less delayed gastric emptying, less transfusion, faster postoperative recovery, and shorter hospital stay compared with conventional open pancreatoduodenectomy (OPD), benefiting from the breakthroughs of laparoscopic instruments and technologies [3–7]. Nevertheless, because it is complex and time-consuming, LPD is likely to tire surgeons, making it an unappealing treatment option.

Giulianotti et al. reportedrobotic pancreatoduodenectomy (RPD) for the first time in 2003, and it has since played a revolutionary part in the advancement of minimally invasive PD [8]. RPD can dramatically overcome some limitations of LPD by providing excellent ergonomics, instrument joints with 540° of movement, automatic tremor removal, and generation of high-definition of 3D images at 10–15 × magnifications, all of which aid in the delicate and complex procedure and reduce the surgeon’s fatigue [9, 10]. However, RPD has not been adopted universally as a result of the high expense of the instrument, technical challenges, risks of surgery, and oncological outcomes [9–12].

This study focuses on exploring the safety, feasibility, and justification of RPD by comparing the perioperative outcomes among OPD, LPD, and RPD groups based on PD data from the same pancreatic treatment team at the First Affiliated Hospital of Guangxi Medical University over the same time period.

## Methods

### Patients and data setting

**Fig. 1:**
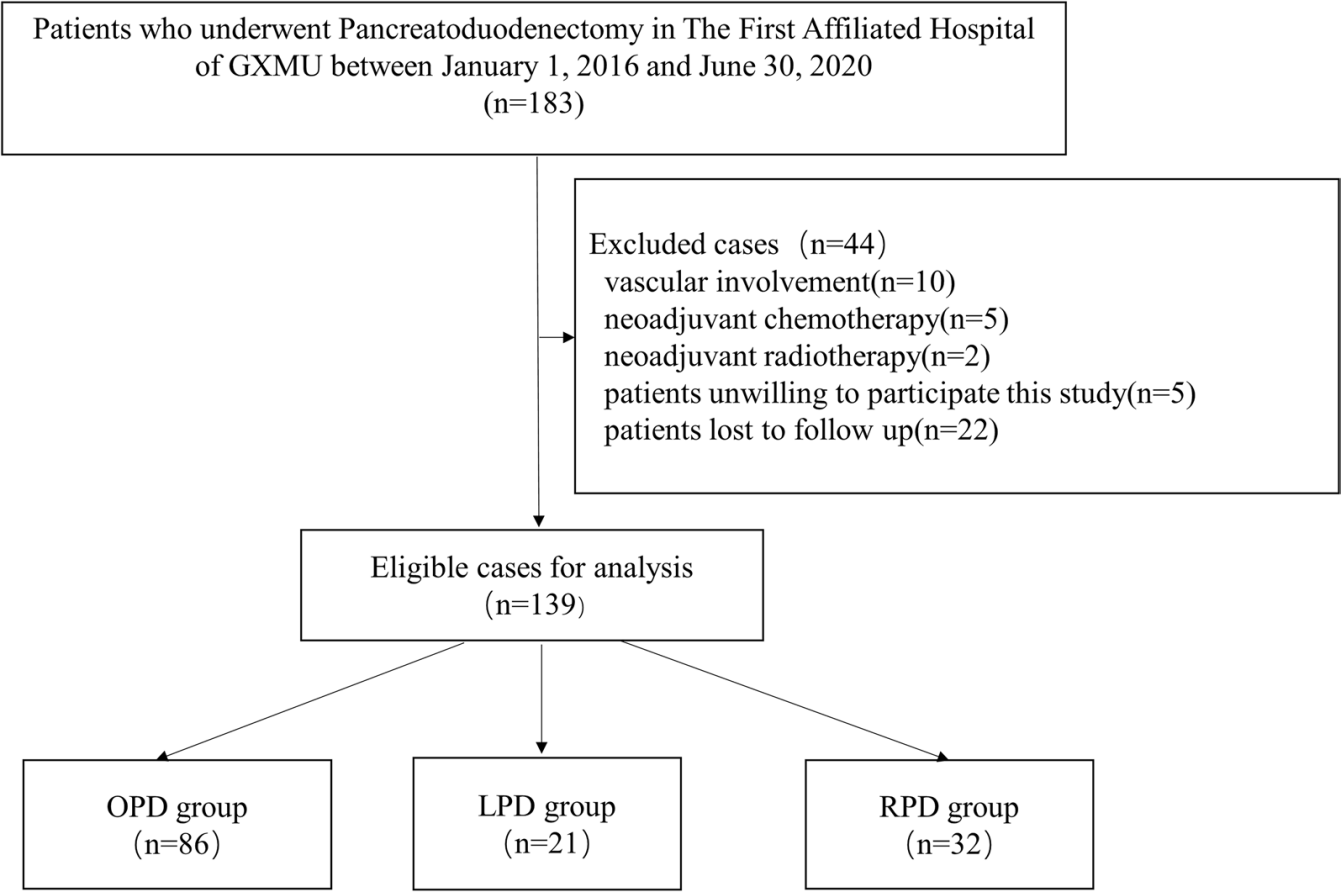
Study flow diagram

From January 2016 to June 2020, the medical records of 183 patients who underwent PD procedures by three skilled surgeons with many years of training and who perform more than 20 PDs each year were retrospectively analyzed. To characterize patients consistently, we included the patients who underwent PD for benign, premalignant, or malignant indications. Exclusion criteria were as follows: (1) vascular involvement, (2) receipt of neoadjuvant chemotherapy or neoadjuvant radiotherapy before operation, (3) concomitant resection of another organ, and (4) patients unwilling to participate in this study or lost to follow up (Fig. 1).

On admission, demographic and anthropometric data and a detailed clinical history were recorded, followed by laboratory and radiological investigations. The major laboratory data and their normal ranges were total bilirubin (TBil): 3.4–20.5 μmol/l, albumin (ALB): 40–55 g/l and hemoglobin (Hb): 130–175 g/l.

### Surgical methods

The details of our surgical procedure have been described in previous literatures [13–16]. The bile duct, duodenum, head of pancreas and uncinate process were mobilized and dissected successively. In the construction phase, we performed retrocolic end-to-side pancreatojejunostomy with a short internal stent and absorbable sutures (PDS II 4–0 or 5–0), retrocolic end-to-side hepaticojejunostomy with a running suture of absorbable 4–0 barbed suture, and antecolic side-to-side gastrojejunostomy with a linear cutter stapler.

Selection criteria for LPD and RPD were (1) patients in generally good condition, (2) capable of tolerating pneumoperitoneum for a long time, (3) without serious obesity (defined by a body mass index (BMI) ≥ 40), and (4) without vascular involvement or proximity to main vessels. LPD or RPD were converted to OPD under the following circumstances: (1) the intraperitoneal adhesions were extensive and dense, and it was difficult to separate under laparoscopy, resulting in more bleeding; (2) the tumor was large and the operative field exposure was poor; (3) intraoperative blood loss reached 1000 ml, which is the warning line for conversion to OPD; (4) blood loss exceeded 1500 ml and the operation was unable to be completed; or (5) uncontrollable sudden and massive bleeding occurred.

### Definitions of surgical risks

Surgical risks were as follows: perioperative mortality, that is, death within 90 days after surgery or during hospitalization; postoperative pancreatic fistula (POPF) grades B and C, based on the new definition and grading system of the International Study Group for Pancreatic Fistula (ISGPF) [17]; post-pancreatectomy hemorrhage (PPH) [18], as defined by the International Study Group of Pancreatic Surgery (ISGPS); bile leakage, as defined by the International Study Group of Liver Surgery (ISGLS) [19]; intra-abdominal infection (IAI), as defined by the Surgical Infection Society (SIS) [20]; activity of daily living (ADL), measured according to the Barthel index scale; and pain, scored using was in the Wong-Baker FACES Pain Rating Scale (https://wongbakerfaces.org/).

Medical complications were defined according to the Clavien-Dindo classification [21], such as pneumonia (grade II), noninfectious diarrhea (grade I), anastomotic leakage (grade IIIb), biloma (grade IIIa) and wound infection (grade I, grade II, grade IIIa), etc. The treatments for these complications include antibiotics, percutaneous drainage or relaparotomy, early irrigation, and debridement.

### Management of complications

Management of POPF: For grade B POPF, the management plan in our center is as follows. I. Drainage. The treatment of pancreatic fistula is based on unobstructed drainage. When drainage is not smooth, interventional ultrasound or CT-guided puncture drainage should be adopted. II. Infection control. Use broad spectrum antibiotics according to experience, keep the drainage fluid for culture, and adjust the use of antibiotics according to the results of drug sensitivity test. III. Nutritional support. Improving nutritional status is conducive to the healing of pancreatic fistula, and attention should be paid to the control of blood glucose, correction of hypoproteinemia and anemia, and maintenance of water and electrolyte balance. For Grade C POPF, surgical treatment should be considered when non-surgical treatment is ineffective.

Management of PPH: Mild bleeding can be considered as a non-surgical treatment (such as application of hemostatic drugs), and the patient's clinical manifestations should be closely observed. For moderate to severe abdominal bleeding, surgical treatment is recommended. If gastrointestinal bleeding is suspected, vascular intervention, endoscopy and other treatments can be selected according to technical conditions, and surgical hemostasis should be actively performed if necessary.

Management of bile leakage: Drainage, infection control, and nutritional support should be administered.

Management of IAI: Ultrasound or CT-guided percutaneous catheterization is recommended as the first choice within 24 h after diagnosis of intraperitoneal infection and initial empiric therapy should be administered with a broad-spectrum antibiotic. For severe gastrointestinal fistula or anastomotic fistula complicated with diffuse intraperitoneal infection, abscess puncture and drainage usually are not good or conservative treatments are often ineffective, laparotomy and external drainage or jejunostomy should be adopted according to the patient's situation.

### Statistical analysis

Statistical analyses were conducted using IBM SPSS Statistics v25. Continuous data were presented as the mean ± standard deviation or the range. Continuous parametric data of three group comparisons were analyzed through one-way ANOVA with Bonferroni correction for multiple comparison. Kruskal- Wallis was adopted to compare continuous nonparametric data. Chi-squared tests or Fisher’s exact test were performed for categorical data. A two-tailed significance level of p < 0.05 was chosen.

## Results

### Patients and baseline characteristics

After screening based on our inclusion criteria, a total of 139 patients were enrolled in this study. The demographics of the three groups are included in Table 1. There were no statistical differences in preoperative clinical parameters, including age, sex, BMI, tumor size, TBil, ALB, Hb, malignant tumor and benign tumor, among the OPD, LPD, and RPD groups.

**Table 1:** Baseline characteristics of the patients included

	OPD (n = 86)	LPD (n = 21)	RPD (n = 32)	*P*
Male	49	12	21	0.690
Female	37	9	11	
Age	57.7 ± 12.3	52.1 ± 13.5	53.7 ± 14.4	0.119
BMI	21.5 ± 3.0	22.6 ± 2.3	21.7 ± 3.0	0.314
Tumor size	3.4 ± 2.1	3.7 ± 2.7	3.4 ± 1.9	0.916
Tbil	94.2 ± 115.0	86.2 ± 97.9	64.8 ± 70.0	0.395
Alb	37.3 ± 3.9	37.5 ± 3.8	37.4 ± 3.8	0.970
Hb	109.9 ± 18.5	114.2 ± 16.9	116.7 ± 21.4	0.259
Malignant tumor	66	17	22	0.572
Benign tumor	20	4	10	

*BMI* body mass index, *Tbil* total bilirubin, *Alb* albumin, *Hb* hemoglobin

### Perioperative outcomes

Perioperative outcomes are summarized in Table 2, postoperative complications in Table 3. Notably, no statistical differences were found regarding blood loss, blood transfusion, length of hospital stay, lymph node yield, or postoperative complications, such as PPH, bile leakage, IAI, POPF, reoperation and perioperative mortality. Although RPD required more operation time than OPD (537.2 min vs. 441.5 min, p < 0.001), the time to remove the abdominal drainage tube was considerably earlier for RPD (12.5 d vs. 17.3 d, p = 0.001). Compared with LPD, RPD had similar operative duration (537.2 min vs. 592.9 min, p = 1.000) and blood loss volume (482.8 ml vs. 559.5 ml, p > 0.05), but higher ADL on postoperative day 3 (35.8 vs. 25.7, p = 0.0017) and a lower rate of conversion to OPD (6.5% vs. 38.1%, p = 0.011). The higher ADL on postoperative day 3 was likely influenced by the lower conversion rate to OPD, but in the subgroups of LPD and RPD cases not converted to OPD, the RPD subgroup still had higher ADL on postoperative day 3 (32.5 vs. 23.0, p = 0.013).

**Table 2:** Perioperative outcomes

	OPD	LPD	RPD	*P*	*p1*	*p2*	*p3*
	n = 86	n = 21	n = 32				
Operation time	441.5 ± 119.8	592.9 ± 204.7	537.2 ± 26.7	0.000	0.001	0.000	1.000
Blood loss	531.7 ± 555.1	559.5 ± 427.1	482.8 ± 663.4	0.053			
Blood transfusion	640.4 ± 794.3	401.4 ± 198.7	490.9 ± 852.1	0.343			
Conversion to open	NA	8 (38.1)	2 (6.5)	0.011			
Lymph node yield	9.0 ± 8.5	9.0 ± 9.4	6.5 ± 10.7	0.913			
Abdominal drainage tube removal	17.3 ± 8.9	18.9 ± 13.9	12.5 ± 6.3	0.002	1.000	0.001	0.177
Catheter removal	3.1 ± 1.7	2.3 ± 1.4	2.7 ± 1.8	0.047	0.031	0.096	0.518
ADL on postoperative day 3	28.9 ± 12.0	25.7 ± 7.1	34.8 ± 16.3	0.046	0.236	0.064	0.017
Pain Score on postoperative day3	1.4 ± 0.9	1.5 ± 0.6	1.1 ± 0.8	0.158			
Length of stay	31.2 ± 12.2	30.0 ± 13.7	28.8 ± 10.1	0.502			

*ADL* activity of daily living Scale, *p1* p value for OPD compared to LPD, *p2* p value for OPD compared to RPD, *p3* p value for LPD compared to RPD

**Table 3:** Postoperative complications

	OPD	LPD	*p1*	RPD	*p2*	*p3*
	(n = 86)	(n = 21)		(n = 32)		
POPF (B/C)						
B	17 (19.8%)	2 (9.5%)	0.434	3 (9.4%)	0.181	1.000
C	2 (2.3%)	1 (4.8%)	0.484	3 (9.4%)	0.240	0.928
PPH	6 (7.0%)	1 (4.8%)	1.000	3 (9.4%)	0.963	0.928
Bile leakage	17 (19.8%)	3 (14.3%)	0.791	4 (12.5%)	0.359	1.000
IAI	17 (19.8%)	4 (19.0%)	1.000	6 (18.8%)	0.901	1.000
Reoperation	5 (5.8%)	2 (9.5%)	0.901	2 (6.2%)	1.000	1.000
Perioperative mortality	2 (2.3%)	0 (0)	1.000	1 (3.1%)	1.000	1.000

*POPF* postoperative pancreatic fistula, *PPH* post-pancreatectomy hemorrhage, *IAI* intra-abdominal infection, *p1* p value for OPD compared to LPD, *p2* p value for OPD compared to RPD, *p3* p value for LPD compared to RPD

## Discussion

RPD has been used in removing various benign and malignant tumors from the head of the pancreas and the periampullary region in recent years, but its adoption remains limited to a few large academic centers, so the availability of associated data is extremely poor. There is no general consensus on the feasibility and safety of RPD and whether it increases surgical risks.

In this study, the RPD, LPD, and OPD groups were fairly similar with regard to most baseline information, such as age, sex, BMI, and tumor size. Accordingly, we believe that the comparison of outcomes among the groups is reliable. Of note, no statistically significant difference in operative time was observed between RPD and LPD, which agrees with the result reported by Zimmerman et al. [22]. Moreover, we noticed that OPD required the least operative time and RPD required the most operative time. The high operative time of RPD was caused by technical difficulties such as the installation and adjustment of robotic arms, especially when the assistants and/or scrub nurses were not well trained or unacquainted with the setup. As robotic technology is developed, the operating time of RPD should be remarkably reduced in the future. We found that blood loss was lower in the RPD group than the OPD and LPD groups, but the difference was not statistically significant. This was similar to the results of Shi et al., who demonstrated that RPD was superior to OPD in estimated blood loss (mean: 297.3 ml vs. 415.2 ml), with a statistically significant difference (p = 0.002) [23]. Furthermore, in an American multi-center study, scientists discovered that RPD offered a mean reduction in blood loss of 181 ml compared to OPD [24]. Interestingly, when Orti-Rodriguez et al. compared an LPD group of 147 patients and an RPD group of 284 patients, the estimated blood loss was significantly higher in the RPD group than in the LPD group (median: 346.44 ml vs. 172.93 ml, p < 0.05) [25]. This discrepancy from our results might stem from patient selection bias and a learning curve.

Additionally, a lower incidence of conversion to OPD was found in the RPD group compared with the LPD group (6.5% vs. 38.1%). This has been corroborated by other published series comparing robotic and laparoscopic approaches [26–28]. More specifically, advantages offered by the robotic approach, including 3D visualization and increased flexibility of the instrument, facilitate the completion of complex resection and digestive tract reconstruction when compared with the laparoscopic approach.

A comparative study between an RPD group with 304 cases and an OPD group with 172 cases by Shyr et al. illustrated that 4.8% of all RPD cases were converted OPD. Remarkably, in the first 100 RPD cases, the conversion rate was roughly 10%, but this figure declined to 7% after 200 cases, and 2.9% after 300 cases, which indicated that the accumulation of experience in surgery could be a dependable predictor of conversion rate [29, 30]. It is widely believed that one of the advantages of robotic and laparoscopic approaches is the reduction of inpatient time [31]. In fact, in a trial by Adam et al., no difference was discovered in hospital stay among 7061 RPD, LPD and OPD patients, including 6078 OPD patients, from the National Cancer Database, which is consistent with our results [32]. The probable reason for this finding in our study is that only a small proportion of the hospital stay is paid for by patients, as most of the stay is covered by medical insurance; hence, patients prefer to remain in the hospital until there is no need to worry about their health issues. We think this could be one of the reasons why the length of hospital stay was higher in our center as compared with most centers in other countries. Additionally, in a recent meta-analysis, Doula et al. [33] reported their findings to be inconclusive as to whether LPD or RPD decreased hospitalization stay. Furthermore, in our study, RPD required less time to remove the abdominal drainage tube than the OPD group (12.5 d vs. 17.3 d, p = 0.001), which may be due to the fact that Da Vinci robotic surgery could decrease inflammatory stress response and peritoneal exudate caused by surgical trauma, as reported by Shibata and his co-workers in 2015 [34]. We also found that there was higher ADL on postoperative day 3 in the RPD group than the LPD group (35.8 vs. 25.7, p = 0.0017). The low conversion rate from RPD to OPD may lead to quicker recovery [35], which could be one of the reasons for this result. In our center, the indications for abdominal drain removal are related to the characteristics and volume of drainage fluid, biochemical examination results (amylase, bilirubin), and bacterial culture results, but we need to be cautious about the removal of the abdominal drainage to prevent the catastrophic consequences of delayed biliary or pancreatic leakage, so the time of the abdominal drainage removal should be decided according to the actual situation.

No significant differences were found in the postoperative complications, such as POPF, PPH, bile leakage, reoperation, and perioperative mortality, among OPD, LPD and RPD. Therefore, RPD appeared not to compromise surgical outcomes, although this retrospective study has limitations due to unavoidable selection bias.

Of note, there are some undeniable limitations in this study. This was a single-center retrospective study with a small sample size, and it lacked randomized controlled trials (RCTs). Additionally, the surgeons in this study were likely still in the early phase of the learning curve of RPD. Further high-quality validation studies are urgently needed.

## Conclusions

This study demonstrates that not only is RPD feasible and reliable for the removal of benign and malignant tumors, but it also offers additional benefits in short-term operative outcomes without increasing the surgical risks. In the future, studies involving numerous subjects and large-scale RCTs are required to establish solid conclusions.

## Data Availability

The datasets of the current study are available from the corresponding author upon reasonable request.

## Abbreviations

PD: Pancreaticoduodenectomy
OPD: Open pancreaticoduodenectomy
LPD: Laparoscopic pancreaticoduodenectomy
RPD: Robotic pancreaticoduodenectomy
ADL: Activity of Daily Living Scale
BMI: Body mass index
POPF: Postoperative pancreatic fistula
ISGPF: International Study Group for Pancreatic Fistula
PPH: Post-pancreatectomy hemorrhage
ISGPS: International Study Group of Pancreatic Surgery
ISGLS: International Study Group of Liver Surgery
IAI: Intra-abdominal infection
SIS: Surgical Infection Society
TBil: Total bilirubin
ALB: Albumin
Hb: Hemoglobin
RCTs: Randomized controlled trials
